# Community-Based Testing for SARS-CoV-2 — Chicago, Illinois, May–November 2020

**DOI:** 10.15585/mmwr.mm7019a4

**Published:** 2021-05-14

**Authors:** Kayla English, Uei Lei, Frankie Shipman-Amuwo, Micah Burkey, MSP, José G. González, Sarah Richardson, Maribel Chavez-Torres, M. Allison Arwady, Christina Anderson, Jennifer E. Layden, Peter Ruestow, Massimo Pacilli, Isaac Ghinai

**Affiliations:** ^1^Chicago Department of Public Health; ^2^Epidemic Intelligence Service, CDC.

On May 13, 2020, Chicago established a free community-based testing (CBT) initiative for SARS-CoV-2, the virus that causes COVID-19, using reverse transcription–polymerase chain reaction (RT-PCR). The initiative focused on demographic groups and geographic areas that were underrepresented in testing by clinical providers and had experienced high COVID-19 incidence, including Hispanic persons and those who have been economically marginalized. To assess the CBT initiative, the Chicago Department of Public Health (CDPH) compared demographic characteristics, economic marginalization, and test positivity between persons tested at CBT sites and persons tested in all other testing settings in Chicago. During May 13–November 14, a total of 253,904 SARS-CoV-2 RT-PCR tests were conducted at CBT sites. Compared with those tested in all other testing settings in Chicago, persons tested at CBT sites were more likely to live in areas that are economically marginalized (38.6% versus 32.0%; p<0.001) and to be Hispanic (50.9% versus 20.7%; p<0.001). The cumulative percentage of positive test results at the CBT sites was higher than that at all other testing settings (11.1% versus 7.1%; p<0.001). These results demonstrate the ability of public health departments to establish community-based testing initiatives that reach communities with less access to testing in other settings and that experience disproportionately higher incidences of COVID-19.

Because of limited access to SARS-CoV-2 diagnostic testing in the early phase of widespread transmission in Chicago, CBT sites began operations on May 13, 2020. The City of Chicago’s CBT initiative, with direction from CDPH and the Racial Equity Rapid Response Team,^†^ located sites at community assets (e.g., schools and parks) in areas accessible to Black and Hispanic communities, and in areas with lower per-capita testing rates; testing was offered at no cost to persons tested. These areas were primarily in northwest and southwest Chicago. The CBT initiative focused specifically on Hispanic^§^ persons, because this population had the highest daily incidence of COVID-19 of any racial/ethnic group in Chicago during May 13–November 14, 2020 (*1*). Demographic information was collected during online or on-site registration. No strict eligibility criteria were applied and anyone could seek testing; however, the initiative attempted to give priority to disproportionately affected communities and persons with symptoms, persons who had had close contact with someone with confirmed COVID-19, or persons who had taken part in activities that put them at higher risk for COVID-19. Initially, fixed CBT sites were established. Sites were administered by the Community Organized Relief Effort (CORE),^¶^ which hired English- and Spanish-speaking staff members from local communities to supervise specimen collection, manage site operations, and engage with the community directly.

After overall COVID-19 incidence declined and transmission became increasingly localized, the number of fixed CBT sites were reduced from six to four on June 23, 2020, and a mobile testing strategy was begun. Mobile sites were deployed to zip codes with the highest 7-day average percentage of positive test results. Most mobile sites remained in place for 1–2 days, and many were redeployed more than once to the same location during the study period, if that location continued to have a high percentage of positive test results. CDPH and CORE promoted sites with messages in English and Spanish and partnered with community-based and faith-based organizations to identify and advertise CBT sites. Beginning September 23, 2020, persons seeking testing were asked to show a health insurance card or state identification at registration to allow Chicago to seek reimbursement from health insurance or the Health Resources and Services Administration (HRSA); showing either document was optional.** At all CBT sites, including fixed and mobile sites, oral swab specimens were self-collected under supervision and tested for SARS-CoV-2 by RT-PCR using the Curative SARS-CoV-2 assay.^††^ CDPH provided test results, along with relevant guidance on COVID-19 isolation and quarantine of contacts or prevention of COVID-19, by email or by personal telephone call for persons without a valid email address or who declined email follow-up.

Demographic characteristics, economic marginalization, and percentage of positive test results were compared between persons tested at CBT sites and persons tested at any other setting in Chicago. Characteristics of Chicago residents tested at all other settings were extracted from the Illinois National Electronic Disease Surveillance System.^§§^ Economic marginalization was assessed according to the Intercity Hardship Index (IHI) of the person’s zip code of residence; IHI is a composite measure used to compare the economic condition of cities over time, based on unemployment, dependency, education, income level, crowded housing, and poverty (*2*). The IHI for each Chicago zip code was calculated and tertiles were derived. For this analysis, residents of zip codes with an IHI in the highest or lowest tertiles were defined as experiencing high or low levels of economic marginalization. Pairwise comparisons between groups were assessed using Pearson’s chi-square test. P-values <0.05 were considered statistically significant. Analyses were conducted using SAS (version 9.4; SAS Institute). This activity was reviewed by CDC and was conducted consistent with applicable federal law and CDC policy.^¶¶^

During May 13–November 14, approximately 1.6 million COVID-19 tests were conducted in Chicago, including 253,904 (16%) at CBT sites and 1,346,994 (84%) in all other testing settings. Overall, 11.1% of all SARS-CoV-2 test results at CBT sites were positive, with higher percentages of positive tests at CBT sites than in all other testing settings (11.1% versus 7.1%; p<0.001) (Figure). Differences between the percentage of positive test results at CBT sites and all other testing settings increased from epidemiologic week 29, after overall increases in citywide incidence and increases in mobile testing. Test positivity across mobile and fixed CBT sites was similar (11.1% versus 11.2%, respectively) (Table).

**FIGURE:**
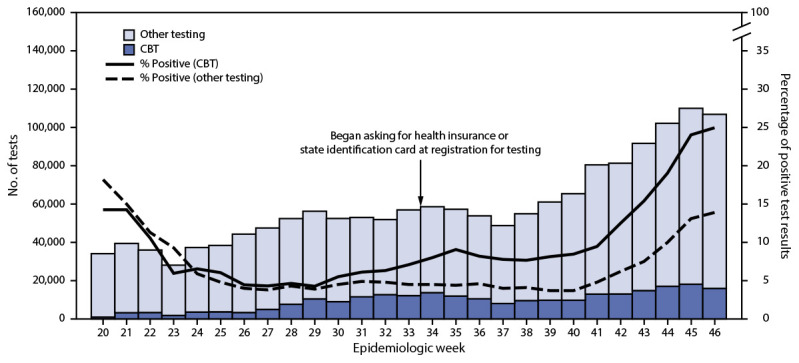
Number of SARS-CoV-2 tests and percentage of positive test results, by test setting and epidemiologic week* — Chicago, Illinois, May 13–November 14, 2020^†^ **Abbreviation:** CBT = community-based testing. * Epidemiologic week is a standardized measure of week, from Sunday through Saturday and ranging from 1 to 52 (sometimes 53), throughout the year; epidemiologic week 20 corresponds to the week beginning May 10, 2020. ^†^ Chicago established a free CBT initiative for COVID-19, which focused on groups underrepresented in testing and with high levels of COVID-19, on May 13, 2020. Other testing includes Chicago residents tested in all other settings, as reported through the Illinois National Electronic Disease Surveillance System.

**TABLE:** Characteristics of persons receiving SARS-CoV-2 testing at community-based testing sites compared with those in all other settings — Chicago, Illinois, May 13–November 14, 2020

Characteristic	No. (%)				p-value^¶^
	Mobile CBT sites*	Fixed CBT sites^†^	Total CBT sites	All other settings^§^	
**Total**	**57,828**	**196,076**	**253,904**	**1,346,994**	**—**
**Age group, yrs**					
0–17	7,427 (12.8)	22,789 (11.6)	30,216 (11.9)	85,375 (6.3)	<0.001
18–29	17,154 (29.7)	62,419 (31.8)	79,572 (31.3)	340,965 (25.3)	
30–39	12,058 (20.9)	48,094 (24.6)	60,152 (23.7)	270,978 (20.1)	
40–49	8,011 (13.9)	27,222 (13.9)	35,233 (13.9)	181,798 (13.5)	
50–59	6,100 (10.6)	19,113 (9.8)	25,213 (9.9)	176,079 (13.1)	
60–69	4,513 (7.8)	10,970 (5.6)	15,483 (6.1)	150,340 (11.2)	
≥70	2,539 (4.4)	5,396 (2.8)	7,935 (3.1)	140,157 (10.4)	
Unknown	26 (0)	74 (0)	100 (0)	1,302 (0.1)	
**Sex**					
Female	32,799 (56.7)	108,623 (55.4)	141,422 (55.7)	716,631 (53.2)	<0.001
Male	24,655 (42.6)	85,693 (43.7)	110,348 (43.5)	581,671 (43.2)	
Other	374 (0.7)	1,760 (0.9)	2,134 (0.8)	—	
Unknown	—	—	—	48,692 (3.6)	
**Race/Ethnicity**					
Asian, NH	1,451 (2.5)	6,251 (3.2)	7,702 (3.0)	40,752 (3.0)	<0.001
Black, NH	9,979 (17.3)	29,276 (13.4)	36,255 (14.3)	222,823 (16.5)	
Hispanic	28,773 (49.8)	96,158 (49.0)	124,931 (49.2)	152,701 (11.3)	
Other, NH	3,267 (5.7)	10,693 (5.5)	13,960 (5.5)	50,025 (3.7)	
White, NH	12,747 (22.0)	49,644 (25.3)	62,391 (24.6)	271,510 (20.2)	
Unknown	1,611 (2.8)	7,054 (3.6)	8,665 (3.4)	609,183 (45.2)	
**Test result**					
Positive	6,391 (11.1)	21,915 (11.2)	28,306 (11.1)	96,036 (7.1)	<0.001
Negative	50,717 (87.7)	171,222 (87.3)	221,939 (87.4)	1,244,279 (92.4)	
Indeterminate	720 (1.2)	2,939 (1.5)	3,659 (1.4)	6,679 (0.5)	
Economic marginalization**					
Low	8,499 (14.7)	38,396 (19.6)	46,895 (18.5)	354,795 (26.3)	<0.001
Medium	17,790 (30.8)	64,817 (33.1)	82,607 (32.5)	505,253 (37.5)	
High	24,615 (42.6)	73,417 (37.4)	98,032 (38.6)	431,417 (32.0)	
Unknown	6,924 (12.0)	19,446 (9.9)	26,370 (10.4)	55,529 (4.1)	

**Abbreviations:** CBT = community-based testing; IHI = Intercity Hardship Index; NH = non-Hispanic.

* Selected weekly to specifically target zip codes with high or increasing incidence of COVID-19.

^†^ Fixed locations that operate in communities with reduced access to SARS-CoV-2 testing.

^§^ Includes academic and community hospitals, congregate settings, federally qualified health centers, private providers, pharmacies, and all other testing sites.

^¶^ p-values are for a chi-square test for a global difference in a characteristic between all those tested at total CBT sites and those Chicago residents tested in all other settings as reported through the Illinois National Electronic Disease Surveillance System.

** Calculated based on the IHI of a person’s zip code; residents of zip codes with an IHI in the highest tertile among all Chicago zip codes were defined as experiencing high levels of economic marginalization, and residents of zip codes with an IHI in the lowest tertile were defined as experiencing low levels of economic marginalization. https://data.cityofchicago.org/api/assets/A02C1C5F-8D89-466C-8492-B1FED3DA4C87

Compared with persons tested in all other settings, those tested at CBT sites were more likely to be aged <40 years (66.9% versus 51.7%; p<0.001) (Table). Race and ethnicity data were less likely to be missing for persons tested at CBT sites than for persons tested in all other settings (3.4% versus 45.2%; p<0.001). Among those with known race and ethnicity, persons tested at CBT sites were more likely than were those tested in all other settings to be Hispanic (50.9% versus 20.7%; p<0.001) and, based on zip code IHI, to have experienced high levels of economic marginalization (38.6% versus 32.0%; p<0.001). The proportion of persons tested at CBT sites who identified as Hispanic remained high even after health insurance information started to be collected (46.5% after September 23 versus 48.0% before). Persons tested at mobile sites were demographically similar to those tested at fixed sites; however, those tested at mobile sites were more likely than were those tested at fixed sites to live in a zip code experiencing economic marginalization (42.6% versus 37.4%; p<0.001).

## Discussion

During May 13–November 14, 2020, approximately 1.6 million COVID-19 RT-PCR tests were conducted in Chicago, including approximately 250,000 (16%) through the city’s CBT initiative. The CBT initiative effectively reached communities disproportionately affected by COVID-19, including Black and Hispanic communities and persons living in zip codes with high levels of economic marginalization (*3*,*4*). Mobile sites were particularly effective in reaching persons living in economically disadvantaged neighborhoods. The identification of persons with COVID-19 through the widespread availability of testing for persons with symptoms or those who have had close contact with persons known to have COVID-19 is critical, and consistent with CDC recommendations to contain the spread of COVID-19 (*5*). To advance health equity, such efforts are particularly important among populations disproportionately affected by COVID-19 and with less access to diagnostic testing through other means.

Although there were concerns that collecting health insurance information or identification might dissuade those in the highest risk groups, including undocumented persons, from using CBT sites, the proportion of Hispanic persons seeking testing remained similar after sites started collecting this information. Seeking reimbursement through health insurance or HRSA might relieve the economic impact on public health departments and allow jurisdictions to sustain these operations while preserving equitable access. In Chicago, a community engagement team, including the city’s Racial Equity Rapid Response Team, CDPH, CORE, and other partners, helped guide CBT efforts. This partnership between community-based organizations and government might represent a replicable model to mitigate inequities in access to other health services.

The findings in this report are subject to at least five limitations. First, large amounts of demographic data are missing, particularly the race and ethnicity of those who sought testing outside of CBT sites (45.2% missing). However, in a separate, unpublished CDPH study, missing race and ethnicity data were imputed using probabilistic methods based on individual persons’ last name and U.S. Census tract of residence. This imputation did not materially change the general distribution of race and ethnicity in the sample.*** Second, the extent to which publicly funded CBT is additive by serving persons who would not have otherwise been tested, rather than partly replacing clinical testing, is not well understood. Third, although the proportion of persons identifying as Hispanic remained similar after collection of insurance information or identification began, these changes coincided with intensifying efforts to attract Hispanic communities through intentional messaging and enhanced Spanish-language media; these efforts might have offset possible declines that might have occurred in their absence. Fourth, although the proportion of positive test results was higher at CBT sites compared with that in all other settings, testing in other settings included high-volume testing of low prevalence groups (e.g., university students), whereas CBT deliberately located mobile testing sites in zip codes with high percentages of positive test results. Finally, dynamics of race, ethnicity, economic marginalization (*6*), and COVID-19 (*7*) in Chicago might not be generalizable to other jurisdictions.

This study demonstrates the capacity of public health agencies to establish community-based testing sites that reach communities disproportionately affected by COVID-19 and that have less access to testing in other settings. Collaboration between public health entities and community-based organizations is integral to promoting equitable access to affordable COVID-19 testing (*8*). The Advisory Committee on Immunization Practices has highlighted mitigating health inequities as an important ethical principle in distributing COVID-19 vaccines (*9*). Collaborative models developed through establishing community-based testing could be leveraged in this forthcoming effort.

**Summary**

**What is already known about this topic?**

Chicago established a free community-based testing (CBT) initiative for COVID-19, focusing on groups underrepresented in testing and who experienced high levels of COVID-19.

**What is added by this report?**

During May 13–November 14, 2020, a total of 253,904 tests were conducted at CBT sites. Compared with persons in other testing settings, those tested at CBT sites were more likely to be Hispanic and to live in areas that are economically marginalized. The proportion of positive test results was larger at CBT sites.

**What are the implications for public health practice?**

CBT initiatives led by public health departments can reach communities with less access to testing in other settings and disproportionately higher COVID-19 rates.

## Footnotes

* These authors contributed equally to this report.

†
https://www.chicago.gov/city/en/depts/mayor/press_room/press_releases/2020/april/RERRTUpdate.html

§ The registration form collected ethnicity as Latinx rather than Hispanic, but ethnicity is reported as Hispanic per U.S. Census guidelines. https://www.census.gov/topics/population/hispanic-origin.html

¶
https://www.coreresponse.org/about-us

** Providing this information was voluntary. Chicago emphasized in public messages that testing remained at no cost to the person being tested and was accessible regardless of the ability to provide proof of health insurance, identification, proof of residence, or immigration status.

††
https://www.fda.gov/media/137089/download

§§
https://www.dph.illinois.gov/topics-services/diseases-and-conditions/infectious-diseases/infectious-disease-reporting

¶¶ 45 C.F.R. part 46; 21 C.F.R. part 56; 42 U.S.C. Sect. 241(d), 5 U.S.C. Sect. 552a, 44 U.S.C. Sect. 3501 et seq.

***
https://www.ncbi.nlm.nih.gov/pmc/articles/PMC3922477/pdf/hesr0049-0268.pdf
